# The Acute and Longer-Term Effects of Cold Water Immersion in Highly-Trained Volleyball Athletes During an Intense Training Block

**DOI:** 10.3389/fspor.2020.568420

**Published:** 2020-10-19

**Authors:** Francisco Tavares, Mário Simões, Bruno Matos, Tiaki Brett Smith, Matthew Driller

**Affiliations:** ^1^Medical and Performance Department, Sporting Clube de Portugal, Lisbon, Portugal; ^2^Research Centre, Polytechnic Institute of Maia, Maia, Portugal; ^3^Portuguese Volleyball Federation, Porto, Portugal; ^4^School of Health, University of Waikato, Hamilton, New Zealand; ^5^Sport and Exercise Science, School of Allied Health, Human Services and Sport, La Trobe University, Melbourne, VIC, Australia

**Keywords:** ice baths, adaptation, recovery, hydrotherapy, jump performance

## Abstract

**Background:** The use of cold water immersion (CWI) as a recovery strategy following exercise has drawn mixed findings over the last few decades. The purpose of the current study was two-fold; (1) to determine the acute effects of CWI within the training week, and (2) to investigate the longer-term effects of CWI over a 16-day period.

**Methods:** In a randomized, controlled trial, 13 national-level volleyball athletes were allocated to two groups, an experimental (CWI, *n* = 7) and a control group (*n* = 6) during a 3-week national training camp. The experimental group were exposed to a CWI protocol after the last training session of each day (12 CWI sessions). Measures of lower (countermovement jump and squat jump height) and upper-body (medicine ball throw distance) power were collected pre- and post-training camp. Perceptual and neuromuscular performance measures (countermovement jump) were obtained during the training camp.

**Results:** No significant differences between groups were observed for any measure (*p* > 0.05), however, *small* effect sizes were observed between experimental and control groups on day two of weeks one and two. Three weeks of training resulted in a significant decrease in countermovement jump height in the control group. A moderate effect size (*d* = 0.65) was found for countermovement jump performance between the experimental and control groups.

**Conclusion:** Cold water immersion seems to provide little benefit to recovery in the acute setting (within the training week), however, chronically, there was a trend toward a benefit when implementing cold water immersion in well-trained volleyball athletes over 16 days.

## Introduction

Muscle damage resulting from exercise has been shown to be related to a decrease in performance (Jamurtas et al., 2005). The most commonly investigated acute outcome of exercise-induced muscle damage (EIMD) is reductions in strength and power (Jamurtas et al., 2005). In volleyball, athletes often generate intensive eccentric and concentric activities such as jumps and locomotive actions (i.e., acceleration and deceleration) (Polglaze and Dawson, 1992). During a volleyball match, an athlete performs up to 145 jumps per game (Polglaze and Dawson, 1992). Landing from a jump typically results in the lower body muscles contracting eccentrically to decelerate the movement of the body in the negative direction (Horita et al., 1999). As a consequence of these activities, the EIMD results in an increase in perceived muscle soreness and decrease in neuromuscular function (Clarkson and Newham, 1995; MacIntyre et al., 1995). The high values of muscle damage and consequent muscle soreness expected from volleyball competition (and training), will likely generate acute fatigue, resulting in temporary performance decreases (Freitas et al., 2017). At an elite level, volleyball athletes train twice a day, for two or more days consecutively (Freitas et al., 2017), and during volleyball tournaments, it is not uncommon for teams to compete on successive days. Given the short time to recover between training or competition days, an accumulated level of fatigue can be expected throughout a training or competition week (Freitas et al., 2017) that may result in under-performance or lead to undesirable fatigue states (Freitas et al., 2014).

Sport scientists and coaches frequently implement recovery strategies in an attempt to speed up the recovery process, in addition to the standard recovery methods (i.e., sleep, rest and nutrition) (Tavares et al., 2017a). Within different recovery methods, the use of cold therapies, in particular cold water immersion (CWI), are the most investigated recovery strategies (Tavares et al., 2017a). The exposure to cold water decreases skin, core and muscle temperatures (White and Wells, 2013). This reduction in temperature leads to vasoconstriction, which in turn decreases the acute inflammation from muscle damage (Wilcock et al., 2006). Moreover, a reduction in nerve conduction properties and a decrease in muscle spasm and pain are also expected due to reduced tissue temperature (Wilcock et al., 2006). Lastly, the hydrostatic pressure-induced changes in blood flow may promote muscle metabolite removal, consequently improving metabolic recovery from intense exercise (Bishop et al., 2008; Ihsan et al., 2016). Given the high values of muscle damage arising from volleyball training, CWI would seem appropriate to be implemented when the recovery time between sessions is reduced (i.e., <24–48 h) (Tavares et al., 2017a). However, inflammation, muscle soreness and transient decreases in performance are considered an important part of the training and adaptation process (Bishop et al., 2008). This has led some researchers to suggest that post-exercise cold water immersion may blunt chronic adaptations by reducing muscle protein synthesis and therefore limiting muscle mass maintenance and growth (Roberts et al., 2015). Although some studies investigating the longer-term effects of CWI demonstrate a decrease in anabolic signaling (Roberts et al., 2015), recent research has demonstrated a positive effect on performance in cyclists chronically exposed to CWI during a 6-weeks training camp (Halson et al., 2014). Therefore, two main theories have been proposed for the use of CWI: (1) it enables athletes to perform subsequent training sessions with a greater overall training load); (2) it decreases adaptations to training due to a reduction in the anabolic pathways (Halson et al., 2014).

To the best of our knowledge, studies investigating the longer-term effects of CWI in a highly-trained athletic population are limited to two; one study performed in endurance-trained cyclists (Halson et al., 2014) and the other in professional rugby union athletes (Tavares et al., 2019a). In the cycling study, highly trained cyclists were exposed to CWI four times a week during a 21-days intensification phase followed by a 11-days taper. The authors found a likely beneficial effect of CWI between baseline and taper in the mean power decrease between two 4-min cycling bouts. A likely beneficial effect was also observed in the 1-s maximum mean sprint power in the CWI group when compared to the control. In the rugby study (Tavares et al., 2019a), 23 elite male rugby union athletes were randomized to either CWI (10 min at 10°C, *n* = 10) or a passive recovery control (CON, *n* = 13) during 3 weeks of high-volume training. Although no significant differences were observed between CWI and CON for any measure, CWI resulted in lower fatigue markers throughout the study as demonstrated by the *moderate* effects on muscle soreness (*d* = 0.58–0.91) and interleukin-6 (*d* = −0.83) and *small* effects (*d* = 0.23–0.38) on countermovement jump in comparison with CON. Therefore, based on these two previous studies and the promising results regarding some of the measures, it is clear that further research on the longer-term effects of CWI in highly-trained athletes is warranted.

Cold water immersion has been associated with enhanced recovery in various sports (Leeder et al., 2012). In volleyball, research investigating the effects of CWI is limited to one study (Freitas et al., 2017). In their study, Freitas et al. (2017) investigated the effect of CWI during one training week in professional volleyball athletes. Large positive effect sizes were observed between groups for the changes in countermovement jump (CMJ) performance, testosterone to cortisol ratio and IGF1, suggesting positive effects of CWI (Freitas et al., 2017). The fact that the study from Freitas et al. (2017) only lasted for 1 week, does not allow an understanding of what would have happened during subsequent training weeks. Indeed, longer term effects of CWI in these settings are yet to be established.

Therefore, the aims of the current study are twofold: to investigate the acute effects of CWI within the training week, and to investigate the longer-term effects of CWI during a 3-weeks period. Given previous literature on the effects of CWI, particularly the study performed in volleyball athletes from Freitas et al. (2017), we hypothesized that (1) CWI will enhance recovery during the training week in comparison to a control group; (2) The levels of accumulated fatigue will lead to an increase in perceptual and physiological markers of fatigue throughout the duration of the 3-week study, which will be attenuated in the CWI group.

## Materials and Methods

### Participants

**Figure 1:**
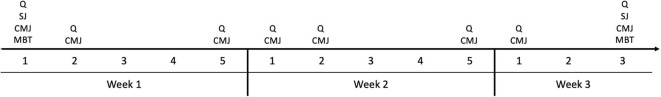
Timeline of study design. Q, questionnaire; SJ, squat jump; CMJ, countermovement jump; MBT, medicine-ball throw.

Fourteen highly-trained volleyball athletes representing the Portugal under-21 national team preparing for qualification to the World Championships, volunteered to participate in the current study (Table 1). Perceived muscle soreness, fatigue, total wellness scores, and neuromuscular performance were collected during a national training camp that lasted for 16 days (Figure 1). Training load was obtained from every training session. Performance measures were collected on day 1 and day 16. In order to be included in the study, participants were required to complete at least 90% of planned training sessions, and not miss more than two training sessions in a row. From the initial sample size (*n* = 14) one athlete failed to meet the inclusion criteria, resulting in 13 athletes completing the study (Table 1). Athletes were matched by positional group (Sheppard et al., 2007) and were randomly divided in one of two groups, a group exposed to 10 min of CWI at the temperature of 10°C with water to the level of the anterior superior iliac spine (*n* = 7; three outside, two middle, one setter, and one libero) and control group (*n* = 6; three outside, one middle, one setter, and one libero). In the experimental group, CWI was performed immediately after the last training session of each training day (totaling 12 CWI sessions). On nine occasions, CWI was performed after the PM training session, and on three occasions, it was following the AM training session. Written informed consent was obtained from each participant, and ethical approval was obtained from the Human Research Ethics Committee of the Institution.

**Table 1:** Participant characteristics.

	**CWI group (*n* = 7)**	**Control group (*n* = 6)**
Age (years)	19.2 ± 0.8	19.0 ± 1.3
Height (cm)	188.1 ± 5.1	187.6 ± 5.6
Body weight (kg)	82.6 ± 8.9	77.4 ± 11.8
Fat mass (%)	10.1 ± 1.9	8.7 ± 0.7
Squat 1-RM (kg)	133.5 ± 20.8	129.2 ± 9.5
Bench Press 1-RM (kg)	72.1 ± 7.1	70.1 ± 10.7

*Data shown as Means ± SD*.

### Procedures

On day 1 and day 16 of the study, athletes were tested for lower and upper body power (Figure 1). During the first 2 weeks, perceptual and neuromuscular performance measures were obtained on day 1, 2, and 5 of each week, while on the last week data was collected on days 1 and 3 of that week (Figure 1). During the experimental period, all training session loads (court and gym-based sessions) from all participants were obtained from individual subjective rating of perceived exertion (RPE). Every athlete attended a meeting with the team nutritionist where they were instructed about meal composition and supplement use. Meal composition and supplement recommendations were equalized for every athlete, but food or diet recall surveys were not conducted. Fat mass was calculated from the three skinfold equation proposed by Evans et al. (2005).

### Training Program

During the first 2 weeks, the athletes performed 12 training sessions per week. For each of the first 2 weeks, moderate to high-load sessions occurred during day 1, day 2, and day 4, while day 3 and day 5 were low- and moderate-load sessions, respectively. During the last week, athletes had a decrease in training load, with a high-load training day on day 1 and low load training day on day 2. The resistance-training program was designed to increase power while maintaining maximum strength (i.e., six exercises of three sets of 6–8 reps with varied loads). There were a total of 29 training sessions (10 resistance training and 19 on-court sessions) throughout the study period.

### Jump Performance

Countermovement jump (CMJ) and squat jump (SJ) tests were used to monitor responses to training. Both jump tests were performed on the morning of the first training day and last training day of the study. In addition, CMJ was performed on the morning of days 1, 2, and 5 of week 1 and 2, and days 1 and 3 of week 3. Following a standardized warm-up composed of dynamic stretches and movements (e.g., bodyweight squats, bodyweight CMJ's) athletes performed 3 CMJ with ~5 s of interval between each jump. In the first and last training days, athletes perform 3 SJ prior to the three CMJ, with 2 min between jumping conditions. All jumps were performed on the top of a 42-cm contact mat (Chronojump-Boscosystem, Barcelona, Spain). The mat was connected to a microcomputer (Chronopic 3, Chronojump-Boscosystem, Barcelona, Spain), which was then connected to a PC via a USB port with analysis conducted by the manufacturers software (Chronojump-Boscosystem Software, Spain). The jumping height was estimated by means of flight time through a standardized kinematic equation *h* = *t*^2^·*g*/8, where *g* is the gravity acceleration (9.81 m/s^2^) (Bosco et al., 1983). For the CMJ, each trial started with the athletes standing in the top of the contact mat with their knees fully extended and the hands on hips to eliminate the influence of arm swing (Tavares et al., 2017b). Athletes were then instructed to descend to a self-selected countermovement depth and to jump as high and quickly as possible. For the SJ, athletes were instructed to hold a self-selected position with shoulders aligned with the top, or in back, of knee level, as used during their normal training routines. Athletes were required to hold that position for 3 s before jumping as high as possible on the command “3, 2, 1, go” (Tavares et al., 2017b). All jumps were monitored by an experienced strength and conditioning coach and if any countermovement was observed, the trial was discarded and an additional trial was performed. The best trial for the SJ and CMJ, determined by the jumping height, was retained for later analysis. Jumping height calculated with this system was previously demonstrated to be valid and reliable (Pueo et al., 2016).

### Seated Medicine Ball Throw

A seated 3-kg medicine ball throw (MBT) was used to measure upper body power. The athletes seated upright at 90° in a chair to facilitate the optimal trajectory and ensure standardization. Athletes performed a warm-up throw followed by two recorded attempts, with a 1-min rest between each attempt and the best distance was recorded. For each trial, the ready position was assumed with the subject placing the ball against their chest. Instructions were to throw maximally using a concentric only motion. Athletes had to maintain their back in contact with the chair, ensuring their feet remained on the floor. Each attempt was measured using a measuring tape taped to the floor and recorded in meters. A similar protocol has been described previously, and shown good test-retest reliability (ICC > 0.97) (De Groot et al., 2012).

### Perceptual Measures

A wellness questionnaire was completed by all participants on the morning of training days 1, 2, and 5 of the first 2 weeks and days 1 and 3 during the last week. The questionnaire was comprised of five questions and was designed to measure the general muscle soreness (1 = very sore, 5 = feeling great), perceived fatigue (1 = always tired, 5 = very fresh), sleep quality (1 = insomnia, 5 = very restful), stress levels (1 = highly stressed, 5 = very relaxed), and mood state (1 = highly annoyed/irritable/down, 5 = very positive mood) of athletes using a 1–5 Likert scale with 0.5-point increments (Tavares et al., 2017b). A total score for each individual was calculated from the average of the five items.

### Training Load

The individual RPE for each resistance training and volleyball training session was obtained between 15 and 30 min following the completion of the session (Tavares et al., 2017b). The training load was then calculated as the product of the individual session RPE and the duration of the session using the following formula: Training load (sRPE) = RPE (1–10) × duration of the session (min) (Foster, 1998; Tavares et al., 2017b).

### Statistical Analysis

The data collected was analyzed using a XLSTAT 19.02.43965 (AddinSoft, New York, NY, USA). Normality and sphericity assumptions were evaluated with the *Shapiro-Wilk* and *Mauchly's* test, respectively. An ANOVA for repeated measures was used to analyze differences between training days and baseline (day 1). *Post hoc* tests with a *Bonferroni* correction were performed to determine where significant differences were observed. If the repeated measures ANOVA assumptions were not met, or the values were presented on an ordinal scale (wellness questionnaires), a Friedman test was utilized. *Post hoc* analyses were performed using the *Dunn-Bonferroni* test or with the *Bonferroni* procedure according to Conover (2009), if a more conservative approach was deemed necessary. A paired sample *t*-test was used to compare pre- to post-differences in performance markers. From the raw data, changes from baseline for each training day for muscle soreness, perceptual fatigue, total wellness score and CMJ was determined for each athlete and an independent *t*-test or Mann-Whitney test was used to compare between-groups differences for each day. A significance level of *p* < 0.05 was implemented for all statistical tests.

The effect sizes (Cohen's *d*) for pre to post-scores for SJ, CMJ and MBT were calculated to measure the difference in the Δ between CWI and CON using an excel spreadsheet (Hopkins, 2006). The same spreadsheet was used to calculate the interaction of the intervention over time for CMJ, wellness total score, fatigue and soreness (Hopkins, 2006). In addition, within group effect sizes were calculated for each variable of interest. Magnitudes of the standardized effects were interpreted using thresholds of 0.2, 0.6, 1.2, 2 and 4 for *small, moderate, large, very large, and extremely large*, respectively (Hopkins et al., 2009). An effect size of <0.2 was considered *trivial*. Where the 90% confidence limits overlapped *small* (±0.2) positive and negative values, the effect was deemed *unclear*.

## Results

**Figure 2:**
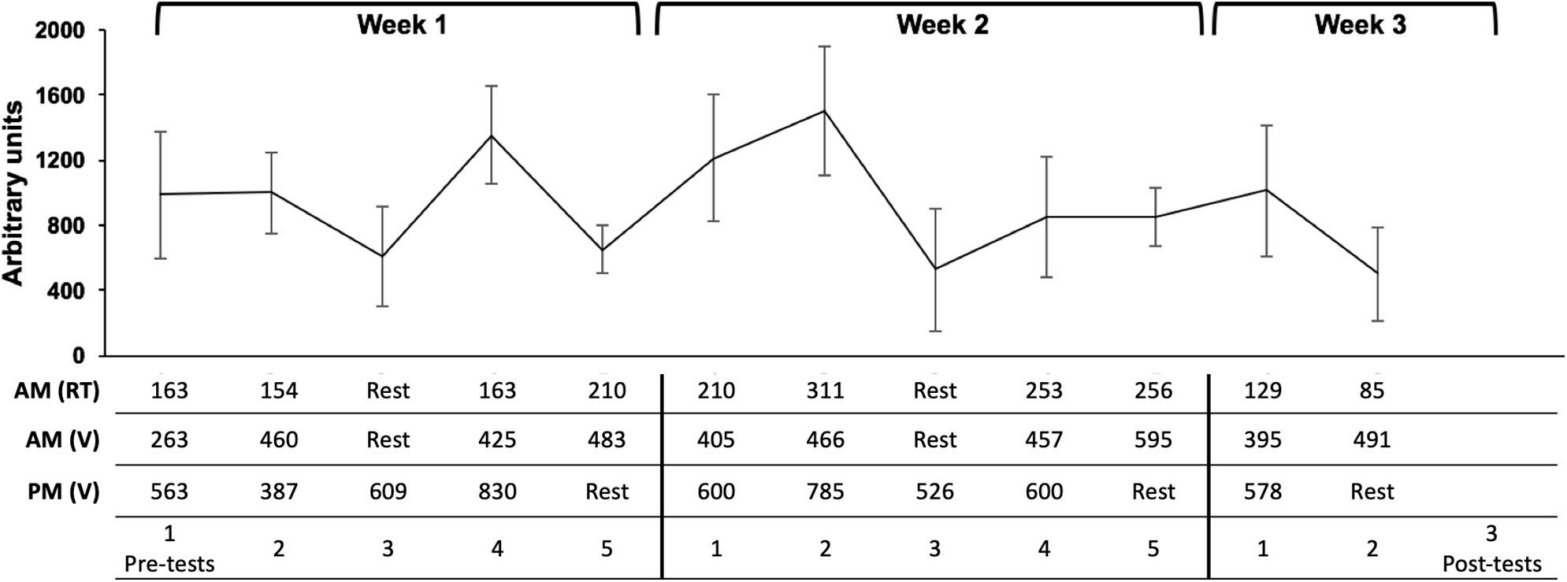
Figure represents mean ± SD daily sRPE (arbitrary units). The table represents the average of the session sRPE (arbitrary units) for each training session. RT, resistance-training session; V, Volleyball training session.

No differences were observed between groups for the athletes' characteristics (Table 1; *p* = 0.051 to *p* = 0.873) or when individual sRPE for each training day were compared (Figure 2; *p* = 0.070 to *p* = 0.697).

**Figure 3:**
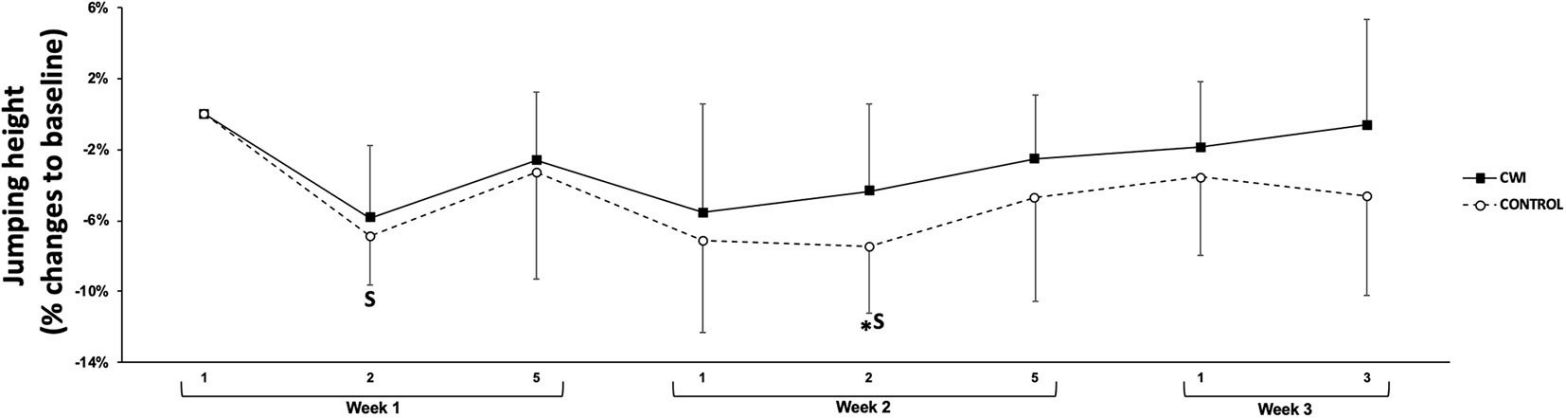
Figure represents countermovement jump height changes to baseline ± SD across time points for CWI and Control groups. *Significant difference (*p* < 0.05) from baseline in the Control group. Effect size (*d*) for between group comparison: S, Small.

Neuromuscular fatigue (from the CMJ) results can be observed in Figure 3. A significant decrease from baseline was observed for control group on the second training day of week two (*p* = 0.049). No differences were observed between groups for any time point (*p* = 0.237 to *p* = 0.812), however, *small* effect sizes were observed between CWI and control (*d* = 0.52) on day two of weeks one and two, favoring CWI.

**Figure 4:**
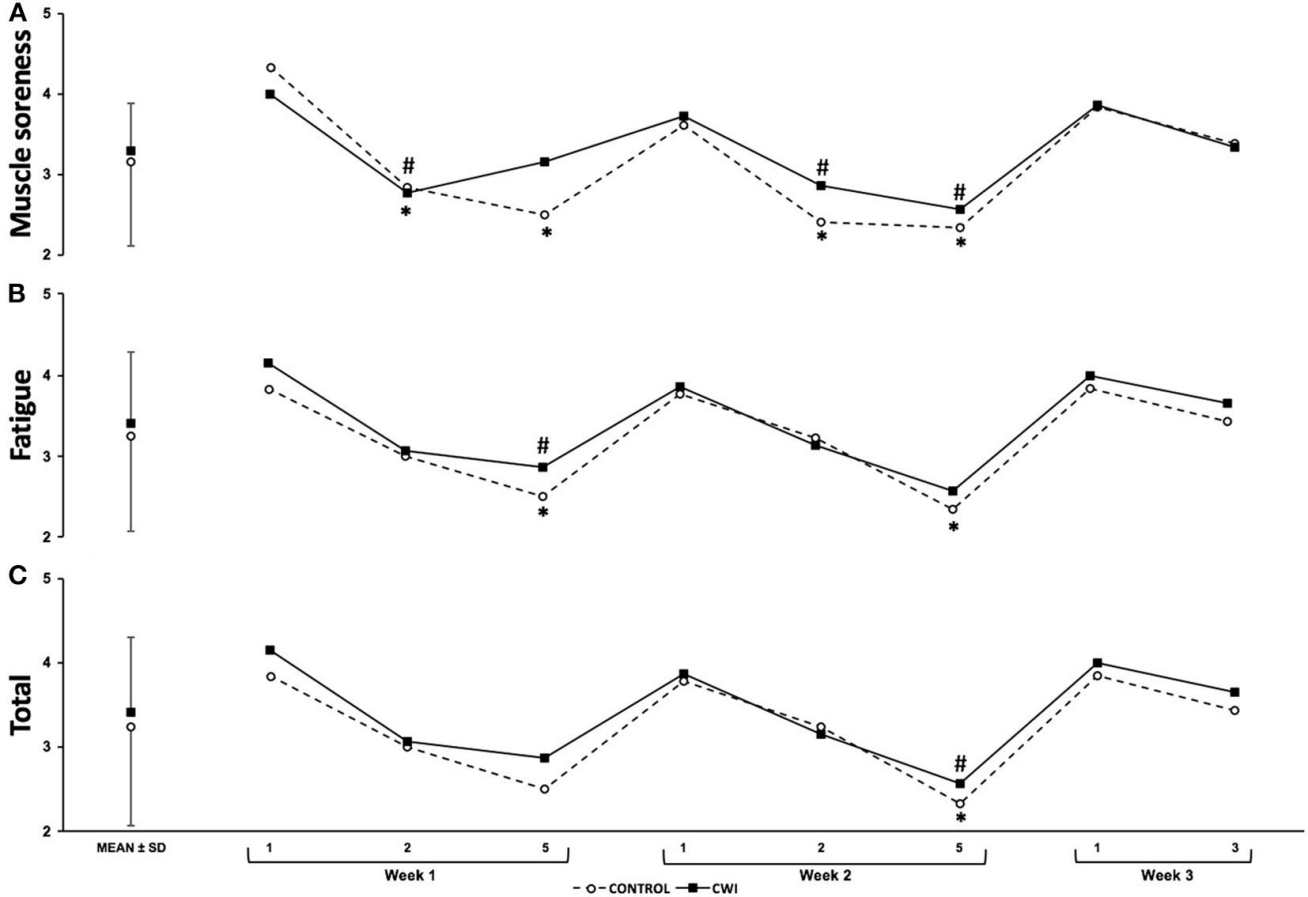
Mean ± SD wellness scores at different time points for CWI and Control groups. **(A)** Soreness; **(B)** Fatigue; **(C)** Total *Significant difference (*p* < 0.05) from baseline in the Control group. ^#^Significant difference (*p* < 0.05) from baseline in the CWI group.

The changes in soreness, fatigue and total wellness scores compared to baseline can be observed in Figure 4. Athletes in the control group were significantly more sore in comparison to baseline on days two and five of week one and two (*p* = 0.001 to *p* = 0.009). Athletes in the CWI group perceived a greater muscle soreness on day two of week one and day two and five of week two (*p* = 0.005 to *p* = 0.038) in comparison to baseline. Perceptual fatigue scores were significantly lower in comparison to baseline in the control group on day five of each week and for the CWI group on day five of week one (*p* = 0.004 to *p* = 0.041). The total wellness score was significantly decreased in comparison to baseline on the last training day of week two in both groups (*p* = 0.005 for control and CWI). Although differences were observed within groups, no significant differences were observed between groups for any training day (*p* = 0.244 to *p* = 1.000). In addition, *unclear* effect sizes were observed in the analysis of the interaction between groups and time for all wellness scores.

No significant differences were found for the CWI or control for the SJ (Table 2; CWI, *p* = 0.309, control, *p* = 0.407). Nevertheless, both groups decreased significantly in the MBT (Table 2; CWI, *p* = 0.005; control, *p* = 0.001). In the CMJ, while the control group decreased significantly (*p* = 0.05), no differences were observed for the CWI group (*p* = 0.408). When the differences of the pre- to post-changes between groups were compared, no significant differences were found for any test (*p* = 0.212 to *p* = 0.976). Nevertheless, *moderate* effect sizes (*d* = 0.65) were found for the CMJ jump between the CWI and control in favor of CWI (Table 2).

**Table 2:** Mean ± SD of performance measures in the CWI and Control groups at different time points, and average of individual change (%) between pre- to post-training.

		**Pre-training**	**Post-training**	**%Δ Post-pre**	**ΔControl–ΔCWI (Effect Size–*d*)**
SJ (cm)	CWI	41.8 ± 2.6	41.0 ± 3.1	−1.6 ± 9.4	−0.9 ± 3.3
	Control	43.0 ± 1.5	43.1 ± 3.6	0.2 ± 5.7	−0.30; *Unclear*
CMJ (cm)	CWI	44.2 ± 1.6	43.9 ± 3.3	−0.6 ± 6.0	2.6 ± 2.7
	Control	49.7 ± 3.4	46.8 ± 4.1^*^	−5.8 ± 5.6	0.65; *Moderate*
Med ball throw (m)	CWI	6.9 ± 0.3	6.0 ± 0.4^*^	−13.0 ± 7.3	−0.2 ± 0.5
	Control	6.6 ± 0.6	6.0 ± 0.7^*^	−10.4 ± 4.1	−0.41; *Unclear*

* *Significant difference (p < 0.05) from pre-training*.

## Discussion

The results from this study are in partial agreement with our original hypothesis which stated that: (1) cold-water immersion would enhance recovery from volleyball training by reducing fatigue levels within the training week; (2) the levels of accumulated fatigue will lead to an increase in perceptual and physiological markers of fatigue, that are attenuated by the use of CWI. The key findings of the study showed that CWI seems to provide little benefit to recovery within the training week. In the longer-term, there was a trend toward a benefit when using CWI in highly-trained volleyball athletes.

In order to understand the acute effects of CWI, results from the first week were analyzed. Both groups significantly increased muscle soreness from day one to day two, however, only muscle soreness in the control group remained elevated on day five (Figure 4). Given the associations between increases in muscle soreness and muscle damage and decreases in muscle function, an increase in fatigue (i.e., perceptual fatigue and neuromuscular performance) was expected (Leeder et al., 2012; Pointon and Duffield, 2012). Although there was a decrease in CMJ between days two (CWI: −5.8 ± 4.0%; control: −6.9% ± 2.8%) and five of week one (CWI: −2.6 ± 3.8%; control: −3.2% ± 6.0%) to baseline, these differences did not reach statistical significance (Figure 3). Nevertheless, a significant decrease from baseline was observed on day five of week one for perceptual fatigue in both groups (Figure 4). This is not surprising as perceptual fatigue is likely to reflect the effect of full body training load rather than only lower body (Tavares et al., 2017b). Although upper body performance was not monitored during the training weeks, the pre- to post-changes observed in medicine ball throw (−12.8 ± 5.8%) support our proposition that perceptual fatigue was significantly higher on day five because of the combination of lower and upper body load.

When the groups were compared, no differences (i.e., individual changes from baseline) in muscle soreness or fatigue were observed between groups on week one (Figures 3, 4). Cold water immersion has been associated with a decrease in muscle soreness and muscle damage markers in various team sports (Tavares et al., 2017a). The high volume of jumps performed in volleyball could potentially result in high values of muscle soreness and fatigue, which may supposedly be attenuated by CWI (Polglaze and Dawson, 1992). In the study from Freitas et al. (Freitas et al., 2017), the authors reported a *small* beneficial effect of CWI on muscle soreness. In addition, *moderate* to *large* effect sizes were observed in biochemical markers measuring endocrine responses, muscle damage and inflammation between groups on the post- to pre-training changes, suggesting a beneficial effect of CWI (Freitas et al., 2017). In agreement with a recent meta-analysis (Leeder et al., 2012), these increases in muscle damage and soreness lead too *small* to *large* effect sizes for CWI enhancing recovery in the Freitas et al. study (Freitas et al., 2017). In the present study, an enhancement in recovery measured by muscle soreness and fatigue (perceptual and physiological) in the group exposed to CWI was expected but not observed. The lack of recorded jumping volumes during training limits our ability to understand if the jumping volume was below the usual expected load, resulting in lower muscle damage and soreness and limiting the effects of CWI. In addition, a low volume, high velocity resistance training programme was predominately implemented during the period of the study. Higher strength training loads are associated with greater muscle disruption (Schoenfeld, 2010) and consequently, higher levels of muscle soreness (MacIntyre et al., 1995), therefore, the limited volume in comparison to other training phases on this type of training may also have limited increases in muscle soreness. Lastly, the fact that muscle soreness was obtained from a single question may mask instances of specific soreness within a particular muscle (e.g., lower body) (Tavares et al., 2017b).

In order to understand the longer-term effects of CWI, perceptual measures and CMJ's were monitored during the second and third weeks. In addition, pre- to post-changes in performance were compared within and between intervention groups. Similar to week one, no significant differences were observed between groups for any of the perceptual measures or for CMJ performance on any of the training days (Figures 2, 3). Nevertheless, in week two, perceptual fatigue and CMJ performance were significantly decreased only in the control group with no significant changes in the CWI group. Moreover, a *small* effect size was observed when comparing the effect of CWI and control groups for CMJ on day two, in favor of the CWI group. This effect of CWI reducing fatigue is further supported by the significant decrease in the pre- to post-CMJ changes observed in the control group but not in the CWI group. In addition, a *moderate* effect size was observed when pre- to post-changes on CMJ were compared between groups (Table 2). The fact that perceptual and neuromuscular fatigue levels were lower in the CWI group, together with the pre- to post-changes in CMJ performance, may demonstrate the efficacy of CWI in reducing the accumulated effects of fatigue. Our results are supported by previous research demonstrating the effects of cold modalities enhancing recovery over prolonged training periods (i.e., 33 days) (Halson et al., 2014). Similarly, the aforementioned study found a beneficial effect of CWI on power output in a highly-trained cyclists (Halson et al., 2014). Research exploring the effects of CWI on performance and perceptual markers of fatigue in team-sport athletes is limited making it difficult to compare our results (Higgins et al., 2012). Although no differences were observed in performance markers between the CWI and control groups obtained from two rugby-specific simulated games separated by 1 week, Higgins et al. (2012) found a trend for beneficial effects of CWI.

As mentioned, previous research has suggested that CWI used in a chronic setting may lead to the blunting of important adaptations, especially in strength and power based sports. For example, Roberts et al. (2015) observed a decrease in the activity of the mammalian target of rapamycin (mTOR) pathway and satellite cells after 10 min of CWI at ~10°C two times a week after resistance training. However, the characteristics of the subjects (recreationally trained) were different from participants in the current study and training load was fixed (e.g., load lifted) so subjects were not allowed to lift heavier even if they felt more *fresh*. Moreover, the training load used in the Roberts study (2 × training sessions per week, compared to 12 sessions a week in the current study) potentially allowed for full recovery between sessions, limiting the rational for the inclusion of cold modalities. The differences in methodologies used (e.g., participants, training frequency, intensity, and duration of CWI) make it difficult to compare between studies. Therefore, we would suggest that further research is needed to provide practical applications to highly-trained team sport athletes, and that understanding the intensity of the training, the density of the week, the athletes' individual goals and the requirements during the season will provide the rational for the implementation of CWI (Tavares et al., 2019b).

Future studies investigating the chronic effect of CWI exposure should monitor training over a longer duration (e.g., >4 weeks) in highly-trained athletes, with high training frequency/load. In addition, given that in volleyball, muscle soreness, damage, and fatigue are likely to be associated with the stretch-shortening cycle activities (e.g., jumps and spikes), these activities should be quantified. Inclusion of tests and questionnaires monitoring upper body neuromuscular performance and soreness may provide important information when exploring the effects of CWI (Tavares et al., 2017b). Finally, while perceptual and mechanical data can provide some information of fatigue and wellness, biochemical measures such as the ones used in the study from Freitas et al. (2017) should be included in future research. This would provide an insight into the mechanisms leading to fatigue and enhance understanding of the effect of CWI in a period where fatigue may be accumulated. We acknowledge that a potential limitation in the current study was the order of tests performed pre and post the study period. The CMJ testing was performed following the SJ testing pre and post study, but not during the training weeks. There is a chance this may have either had a positive effect (e.g., via post-activation potentiation) or a negative effect (e.g., via fatigue) on CMJ performance. However, given the low volume of jumps (3 SJ's) and the time between types of jumps (2 min), we feel it is unlikely to have had a significant impact, and it was also the same for both groups.

Cold water immersion has been shown to enhance recovery (e.g., neuromuscular performance) during periods of accumulated levels of fatigue (Higgins et al., 2012; Halson et al., 2014). In the current study, the effects of CWI were restricted to a *small* effect on CMJ during week 2 and a *moderate* effect on pre to post-CMJ performance. It is however important to mention that the short duration (two and a half weeks) and the fact that training load on week three (i.e., only 2 days) was considerably lower than week one and two, which may have negated a more pronounced effect of CWI on the markers of perceptual and neuromuscular fatigue, soreness and wellness (Figures 2–4). In conclusion, CWI seems to provide little benefit to recovery within the training week. In the longer term, there was a trend toward a benefit when using CWI in highly-trained volleyball athletes. Further research is needed on the use of CWI in highly-trained athletes, implementing longer time-frames and further mechanistic measures to understand what is happening and the muscular level.

## Data Availability Statement

The original contributions generated for the study are included in the article/supplementary material, further inquiries can be directed to the corresponding author.

## Ethics Statement

The studies involving human participants were reviewed and approved by Human Research Ethics Committee, University of Waikato. The patients/participants provided their written informed consent to participate in this study.

## Author Contributions

FT, MS, and BM contributed to the design, data collection, data analysis, and writing of the final paper. MD and TS contributed to the design, data analysis and writing of the final paper. All authors contributed to the article and approved the submitted version.

## Conflict of Interest

The authors declare that the research was conducted in the absence of any commercial or financial relationships that could be construed as a potential conflict of interest.
